# Digital Mental Health Interventions for Young People Aged 16-25 Years: Scoping Review

**DOI:** 10.2196/72892

**Published:** 2025-05-09

**Authors:** Courtney Potts, Carmen Kealy, Jamie M McNulty, Alba Madrid-Cagigal, Thomas Wilson, Maurice D Mulvenna, Siobhan O'Neill, Gary Donohoe, Margaret M Barry

**Affiliations:** 1 School of Psychology Ulster University Coleraine United Kingdom; 2 Health Promotion Research Centre Ollscoil na Gaillimhe – University of Galway Galway Ireland; 3 School of Psychology Ollscoil na Gaillimhe – University of Galway Galway Ireland; 4 School of Psychology Queen's University Belfast Belfast United Kingdom; 5 School of Computing Ulster University Belfast United Kingdom

**Keywords:** digital mental health, youth, mHealth, mobile health, digital intervention, mental health promotion, mental health treatment, well-being, scoping review, PRISMA

## Abstract

**Background:**

Digital mental health interventions for young people offer a promising avenue for promoting mental well-being and addressing mental health issues in this population.

**Objective:**

This scoping review aims to explore the range of digital mental health interventions available for young people aged 16-25 years, with a particular focus on digital tool types, modalities, delivery formats, target populations, and study retention rates.

**Methods:**

The scoping review was conducted in 6 databases (PubMed, Web of Science, Scopus, MEDLINE, Cochrane Library, and PsychInfo). Studies were included if they were published from 2019 to 2024 in English, reported on a population of young people aged 16-25 years, and included validated mental health or well-being outcome measures. All types of digital interventions from promotion and prevention to treatment of mental health were included.

**Results:**

After screening 13,306 articles, 145 articles were included in the final review. The findings reveal a diverse landscape of studies, equally focusing on the prevention and promotion of mental health and the treatment of mental ill health, most commonly using cognitive behavioral therapy (63/145, 43.4%). The most common digital tools were apps (51/135, 37.8%), web-based resources (45/135, 33.3%), and websites (19/135, 14.1%). The results highlight the over emphasis on convenience sampling (140/145, 96.6%), with participants mainly recruited from universities or colleges, and a lack of representation from marginalized groups, including lesbian, gay, bisexual, transgender, and queer youth; those from socioeconomically deprived backgrounds; and those who are neurodivergent. Moreover, the focus on anxiety and depression leaves other mental health conditions underrepresented. Retention rates ranged from 16% to 100% and averaged 66% across all studies.

**Conclusions:**

There is a need for more research on mental health promotion and prevention measures among those aged younger than 25 years as young people are at increased risk of mental health issues. This includes exploring different intervention approaches and modalities beyond cognitive behavioral therapy and ensuring inclusivity in study populations. Standardizing intervention durations and incorporating long-term follow-up data could provide valuable insights into the efficacy and effectiveness of digital interventions. Future studies should aim for greater inclusivity, ensuring representation from marginalized groups to address the diverse mental health needs of young people effectively. By adopting these approaches, digital mental health interventions can become more accessible, engaging, and impactful for young people worldwide.

## Introduction

### Background

In recent years, heightened attention has been directed toward mental health in young people, primarily prompted by the widespread prevalence and profound impact of various mental health conditions. The aggregate estimated prevalence of mental disorders reported for individuals aged 5 to 24 years indicates that more than 1 in 10 children and youth around the world, which equates to 293 million individuals, live with at least one diagnosable mental disorder [1]. The majority of mental disorders appear by the age of 14 years but often remain undiagnosed and untreated in adulthood [2]. The pooled incidence of mental health conditions in children and adolescents has been reported to be 13.4% globally [3], and a recent systematic review found that the incidence rates were higher in Europe at 15.5% [2]. Over the entire life course, 25% of all years lived with disability attributable to mental disorders were recorded before the age of 25 years [1]. The gravity of this matter is striking when looking at various rates of mental ill health around the world [4]. The prevalence of mental disorders varies considerably in different countries, for example, 20% in North America, 12% in Europe and Asia, and 8% in Africa [4]. Differences in gender and age are also present as males are more likely to receive a diagnosis of attention-deficit/hyperactivity disorder compared to females, while females are more likely to be diagnosed with depression compared to males, and adolescents are more susceptible than children to affective disorders and behavior disorders [5]. It is worrying that only 58% of young adults aged 18-25 years with severe mental disorders are accessing treatment [6]. Anxiety, mood disorders, such as depression, and behavioral disorders are among the most prominent contributors [7]. Since 2020, state interventions in the context of the COVID-19 pandemic, such as enforced isolation and school closure, have most certainly increased the burden on young people’s well-being and increased the likelihood of acquiring mental ill health or experiencing worse mental ill health [8]. Lower health-related quality of life and higher anxiety levels are now more common than before COVID-19, particularly among people with a poor socioeconomic position, a migration background, or limited living space [9]. Despite these statistics, the global median of government health spending on mental health is less than 2%, and demand for face-to-face therapy continues to surpass capacity [10,11].

These trends highlight the urgent need for comprehensive strategies to address the complex interplay of mental health challenges using scalable solutions, as reflected in the health policies and strategy reports at both European [12] and global [13,14] levels. The evolution of mental health services to incorporate digital interventions stands out as a promising avenue for effecting positive change on a global scale. One such scalable response has focused on the use of information and communication technology to boost capacity to support and improve young people’s mental health [15].

### Digital Mental Health Interventions

Digital mental health interventions (DMHIs) refer to the digital delivery of well-established psychological treatments by leveraging the use of technology, including digital devices such as computers and smartphones [16]. DMHIs can include text, video, or audio-based technology, and the most commonly used DMHI is internet-delivered cognitive behavioral therapy (CBT) [17]. Internet-delivered CBT has demonstrated high usability and acceptability [18] as well as effectiveness at reducing the symptoms of depression [19] and anxiety [20] among young people.

There are several barriers and facilitators for the use of DMHIs. From the perspective of young people, perceived facilitators include anonymity, accessibility, and prompt feedback [21]. The reported advantages of digital technologies include greater reach to geographically isolated populations, flexible access, increased convenience, fewer visits to specialist clinics, greater privacy and anonymity, enhanced treatment fidelity, rapid scalability, and low-cost delivery [22].

Barriers include lack of personalization to individual needs, fear of misdiagnosis, and issues with the effectiveness of DMHIs [23]. Previous studies have reported low adherence and high dropout rates among adolescents and young adults [24]. Human interaction has been highlighted as an important factor influencing engagement, as low adherence rates have been reported in online interventions without therapeutic guidance [25,26]. Therefore, guided interventions usually have higher engagement rates than unguided interventions [27]. In this context, blended interventions refer to the integration of digital interventions with face-to-face mental health care [28,29], and they have previously been found to be effective in improving students’ mental health [30]. Nonetheless, a previous report has highlighted heterogeneity across meta-analyses in the level of detail regarding the nature of interventions, the target populations, and the type of support delivered, making it challenging to draw strong conclusions with regard to the circumstances under which human support is most effective [31].

### Our Review

Many reviews have been undertaken to evaluate the wide range of digital mental health supports that are accessible, but there is a dearth of up-to-date reviews, with many published in 2021 or earlier, thus failing to consider the change in the digital world since COVID-19 [20,32-35]. Most prior systematic and scoping reviews focused on specific topics only, such as technologies (eg, apps only [36,37]), specific aspects of mental health (eg, depression and anxiety only [20,32,33,35,38] and mental health promotion only [8]), study populations (eg, university students for convenience [38,39]), and research techniques (eg, randomized controlled trials [RCTs] alone [37]). Previous reviews [20,32,33,37-40] primarily focused on determining effectiveness rather than on assessing the implementation features of existing approaches, such as delivery formats, modalities, and retention rates, across the spectrum of interventions from promotion and prevention to early intervention and treatment, which is the focus of this review. Given that previous studies often focused on a single population type, this scoping review explored the inclusion of marginalized populations in the digital mental health support context. UNICEF defines disadvantaged, vulnerable, or marginalized adolescents as “individuals aged 10-19, who are excluded from social, economic, or educational opportunities enjoyed by other adolescents in their community due to numerous factors beyond their control” [41]. These factors include those operating at the social level (eg, economic inequality, violence, stigma, racism, and migration), family level (eg, neglect and abuse), and individual level (eg, disability and ethnicity). Examples of disadvantaged, vulnerable, or marginalized young people include immigrants, refugees, orphans, and those who belong to stigmatized indigenous, ethnic, or religious groups. They also include individuals who identify as belonging to “sexual minorities” (eg, gay, lesbian, bisexual, and queer) or “gender minorities” (eg, transgender and gender diverse), which will be referred to as lesbian, gay, bisexual, transgender, and queer (LGBTQ+) in the context of this review.

The purpose of this study is to report on a scoping review that was undertaken to investigate research findings on the range of DMHIs and supports available for a broad range of young people aged 16-25 years, with a particular focus on digital tools, modalities, delivery formats, target populations, and retention rates.

Specific research questions are as follows:

- What are the characteristics of the studies that have been carried out involving DMHIs for young people?
- What are the characteristics of DMHIs offered to young people and to what degree are young people receiving human support in combination with digital support?
- Who are the main target populations of DMHIs for young people?
- What are the retention rates across studies involving DMHIs with young people?

## Methods

### Protocol

A review protocol for this study has been registered with Open Science Framework (OSF) [42]. The search process was guided by the main stages outlined in the Arksey and O’Malley framework [43]. A search of the literature was initially conducted on December 16, 2022, and updated on June 7, 2024, in the following databases: PubMed, Web of Science, Scopus, MEDLINE, Cochrane Library, and PsychInfo. The search combined terms related to 4 key concepts, including mental health, technology, young people, and interventions. The search strategy can be found in Table 1, and examples of search results can be found in Multimedia Appendix 1. Reporting was guided by the PRISMA-ScR (Preferred Reporting Items for Systematic Reviews and Meta-Analyses Extension for Scoping Reviews) checklist (Multimedia Appendix 2).

**Table 1:** Search terms used to identify studies across 6 key databases.

Topic	Terms (combined together with AND statements)
Mental health	well-being OR wellbeing OR stress OR mental disorder OR mental illness OR mental health
Young people	youth* OR young* OR child* OR adolescen* OR student* OR teen*
Interventions	intervention OR promot* OR prevent* OR program* OR polic* OR implementation OR evaluation OR therap*
Technology	digital* OR mHealth OR eHealth OR web-based OR internet-based OR mobile phone OR text message OR text-based OR SMS OR app OR artificial intelligence OR tele* OR computeri*

### Criteria

Articles published in English only were considered for inclusion. The selection criteria are presented in Textbox 1. In the registered protocol on OSF, the original criteria stated that articles reporting on a study population of those aged 12-25 years would be included; however, this was later refined to 16-25 years. Given that there is no universal agreement on the definition of the age group of youth, populations between 16 and 25 years were selected to capture the out-of-school population of young people in the United Kingdom and Ireland as per education policies, and these ages fall within the range for young people reported by the World Health Organization [44]. Grey literature was not included in the search strategy. Initially, the protocol on OSF stated that the search would include articles from the last 5 years, from 2017 to 2022. However, the search was updated in 2024 and thus included articles published from 2019 onwards. The search was restricted to studies published in the last 5 years (2019-2024) to ensure the review included the most relevant up to date literature, considering the rapid adoption and assessment of digital mental health technologies throughout the sector in recent years, particularly during the COVID-19 pandemic [45] and beyond.

**Selection criteria.**

**Inclusion criteria**

- Population: Young people aged 16-25 years. If the sample population included people aged >25 years or <16 years, the mean age was assessed, and studies with a mean age >16 years and <25 years were included.
- Intervention: Studies that focused on mental well-being, mental health, and mental health conditions, covering all interventions for mental health promotion and prevention, as well as treatment. The focus was on the digital intervention developed or evaluated in the study. Studies with an element of human support were still included if the main intervention component was a digital tool and human support was an adjunct to the digital intervention offered to participants. All types of digital tools were covered, including but not limited to websites, games, computer-assisted programs, chatbots, digital devices, virtual reality, and mobile text messaging.
- Comparator: Not applicable.
- Outcome: Validated pre- and postmental health/well-being outcome measures. Primary or secondary outcome related to mental health.
- Type of publication: Pilot or feasibility studies, randomized controlled trials, nonrandomized controlled trials, other types of randomized trials, longitudinal studies, and mixed methods studies. Studies published in the last 5 years (January 1, 2019, until June 7, 2024).

**Exclusion criteria**

- Population: Young people aged <16 years or >25 years.
- Intervention: Telemental health, telepsychiatry, teletherapy, or telepsychology, including the digital delivery of one-on-one therapy or counseling services, which are traditionally administered in person. In the context of this review, emphasis was placed on delivery or evaluation of a predefined digital mental health intervention program (such as an internet-delivered cognitive behavioral therapy course or other modality) and not on video, phone, or text-based individual counseling or therapy.
- Comparator: Not applicable.
- Outcome: Primary or secondary outcome not related to mental health.
- Type of publication: Qualitative studies, cross-sectional studies, and student theses.

### Study Selection and Data Extraction

All records were imported to Covidence [46]. Duplicates automatically detected by Covidence were removed prior to screening. Title and abstract screening were conducted by 4 independent reviewers (CP, TW, CK, and AMC). During the screening, articles were sorted by title in alphabetical order to compare the title, author name, and abstract with consecutive articles to manually delete duplicates. Three reviewers (CP, TW, and CK) completed the full-text review. For both title and abstract screening and full-text review, 2 independent reviewers screened all articles. Any conflicts were discussed and resolved between these 2 reviewers to reach consensus.

One author (CP) created the data extraction template, which was later refined with 2 additional authors (TW and CK). The data extraction template included the main study characteristics (year, aim, study design, and location), participant details for experimental and control groups (age, gender identity, inclusion of marginalized young people, number of participants, where participants were recruited, method of recruitment [eg, convenience sampling], and participant type [eg, general population]), intervention details (target area, delivery [ie, digital only or blended digital and human support], blended intervention details, type of digital tool, primary and secondary outcome measures, scales used, follow-up, psychological modality used in interventions, duration, incentives, promotion/prevention or intervention, intervention name, and features), and retention details (number of those who started and completed the study). One author (CP) extracted data from all articles included in the scoping review. A second author (CK) completed data extraction for one-third of the articles, and the first author (CP) completed a consensus template for these studies and discussed any discrepancies with the second author.

### Data Analysis

Data were analyzed in R using R version 4.3.2 (R Project for Statistical Computing).

#### Characteristics of the Included Studies

Summaries were computed for each study, including the country of publication, research method, and year of publication. Summary statistics were calculated for the length of studies in weeks and the follow-up length for studies that included a follow-up. Studies were labeled as including a follow-up if outcome measures were collected on any date after the poststudy outcomes were obtained. If studies only recorded pre- and poststudy measures, they were marked as not recording follow-up data. Summary statistics were included for participant numbers in both the experimental and control groups, where reported. Text analysis, using the tm package [47], was performed to explore the most common primary and secondary outcomes, and to measure the most frequent outcomes for validated mental health scales. Summaries were computed for studies that included incentives versus those that did not and for the types of incentives used.

#### Characteristics of Interventions

To explore the characteristics of interventions, the interventions with similar or the same names were checked across studies to identify which studies reported on different aspects of the same digital intervention. A new dataset was created containing the target area for the intervention, the type of digital tool, the features of each intervention, if the intervention was digital only or had an element of human support (blended interventions) and the type of human support (relevant for blended interventions only), and the features of each intervention, with duplicate information removed. Summaries were computed for each of those categories. For intervention features, a list of features was derived based on the intervention description in each article. For example, many articles mentioned that the intervention specifically included psychoeducational content or explained information on mental health and how to manage symptoms, which was also recorded as psychoeducation or education. Some studies referred back to a previous article, which detailed intervention features. Blended interventions were classified as those that had any degree of human support alongside the digital intervention and were categorized according to the author descriptions in the articles. A donut plot was created for the delivery of interventions (digital only or blended) across the different types of digital tools, using the webr package [48]. Given the high degree of overlap within blended interventions, a Venn diagram was used to illustrate the peer, clinician, and research team support offered, and an upset plot (using UpSet R package [49] and ComplexUpset [50]) was used for data visualization of the type of blended support offered.

#### Target Populations

Summaries were computed for target populations across studies. The primary study populations included (1) the general population, (2) those experiencing mental ill health symptoms, and (3) those having a mental health diagnosis. The general population included young people in a general sense with no specific mental health recruitment criteria. The second population most commonly had mild-to-moderate symptoms that were self-reported, but in some cases, they were based on mental health scales, which were administered to determine if participants were experiencing problems with their mental health. The third population had moderate-to-severe symptoms determined either by the participants self-reporting that they had a mental health diagnosis or by researcher-administered mental health scales, where participants received a clinically significant score indicative of a mental health diagnosis (eg, a score of 10 or higher on the Patient Health Questionnaire-9 [PHQ-9] indicated moderate-to-severe depression). Summaries were reported for the sampling approach used, and the recruitment source for participants was visualized using the UpSet R package [49] and ComplexUpset [50]. For participant age across experimental and control groups, the mean of means was calculated from the mean ages reported across all studies that provided this information. To explore gender inclusivity, the gender proportions in each study were explored for those identifying as male, female, and LGBTQ+. Combined boxplots and violin plots were created to visualize the gender identity breakdown, using the ggplot2 package [51]. An upset plot was created to visualize the representation of marginalized groups in study populations across articles, using the UpSet R package [49] and ComplexUpset [50]. Across the 3 main participant types, a donut plot (webr package [48]) was created to illustrate the breakdown of digital-only interventions versus blended human and digital support interventions. Venn diagrams were created to visualize the different psychological treatment modalities, and a grouped bar chart was created to display the proportions of the main intervention features.

#### Retention Rate

There was no way to report on actual engagement with the content within interventions, as studies report this differently or not at all in most cases. Thus, the retention rate was calculated as the number of people that started the study and were allocated to receive the digital intervention divided by the number of people that completed the study, and this was converted to a percentage. For studies that included a follow-up, the number of people that completed the study was taken as the number that completed the final follow-up assessment. For studies that did not include a follow-up, the number of people that completed the study was taken as the number of people that completed the poststudy outcome assessment. Summary statistics were calculated for the retention rate overall and for different categories, including study type, delivery method, type of digital tool, study population, and incentives. Summary statistics of studies with a high retention rate (≥90%) were also calculated in terms of the number of participants recruited, the length of the study, the follow-up measures and when they were recorded, and whether incentives were used.

## Results

### Included Studies

**Figure 1:**
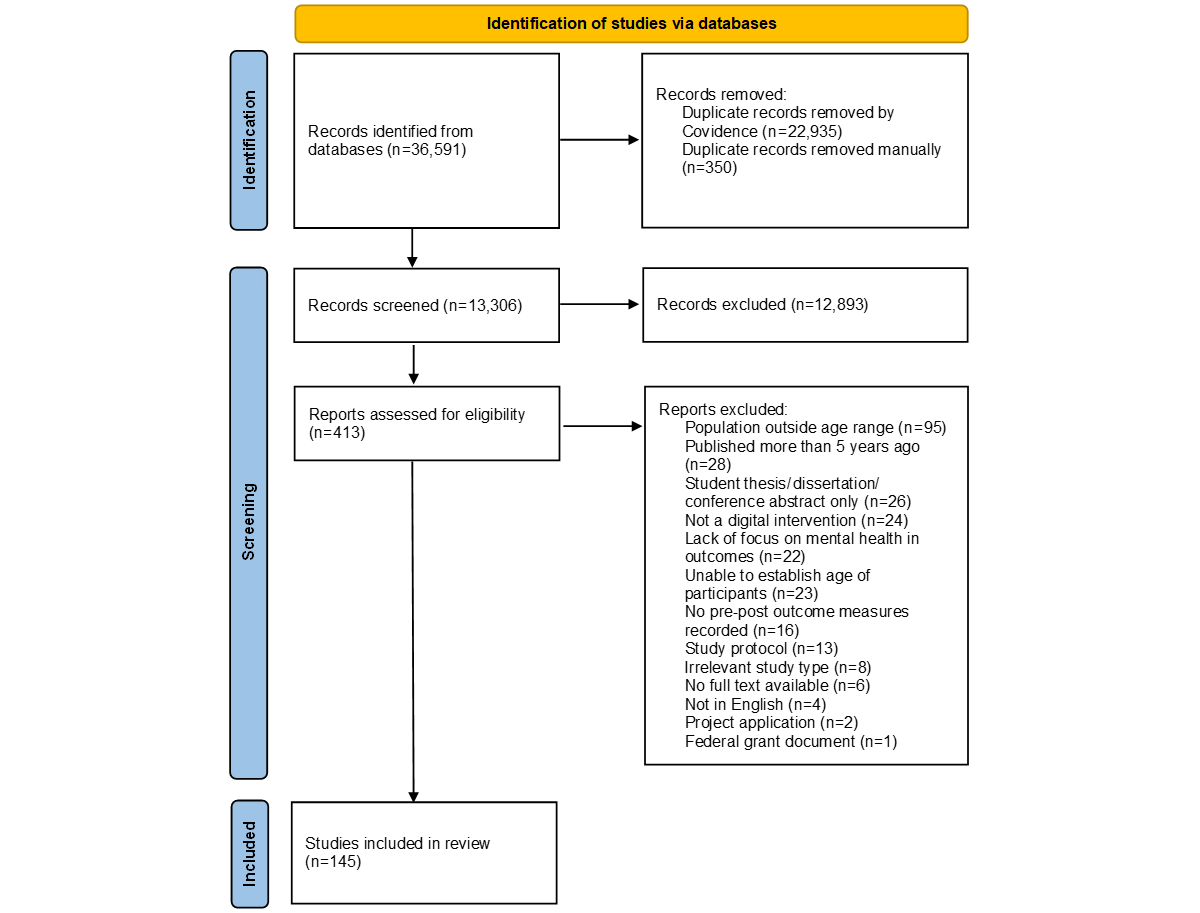
PRISMA (Preferred Reporting Items for Systematic Reviews and Meta-Analyses) flowchart.

A total of 36,591 records were identified across the 6 databases that were searched; however, 23,285 articles were removed as they were identified as duplicates (Figure 1). Through abstract and full-text screening, 12,893 articles were removed, and the reasons for removal are outlined in Figure 1. Eventually, 145 articles were included in the final review (Figure 1).

### Characteristics of the Included Studies

Table 2 provides an overview of the country of origin, research methodology, and year of publication for each study. Of the 145 studies, 80 (55.2%) reported on digital tools for primary prevention or mental health promotion and the remaining 65 (44.8%) reported on digital tools for early intervention or treatment of mental ill health symptoms.

The interventions ranged from one-off single sessions with a digital tool to sessions lasting up to 26 weeks (6 months) (Multimedia Appendix 3). The most common study length was 4 weeks (Multimedia Appendix 3), and the mean study length was 6 weeks. Other prescribed intervention durations were 1-2 weeks, 1-9 weeks, 3-6 weeks, and 3-12 weeks. Two articles did not detail the study length. Less than half of the articles (63/145, 43.4%) recorded participant follow-up outside of the conventional pre-post study data. A total of 48 of these 63 studies recorded only 1 follow-up anywhere from 2 weeks after the intervention period to 52 weeks. The most common follow-up period was 12 weeks, and the mean follow-up period was 19 weeks. Ten studies followed up with participants at 2 time points (eg, at 4 and 12 weeks), and 2 studies followed up with participants at 3 time points (eg, at 8, 12, and 24 weeks).

**Table 2:** Study characteristics.

Characteristic		Value (N=145), n (%)	References
**Country of publication**			
	United States of America	43 (29.7)	[52-93]
	Australia	16 (11.0)	[94-109]
	United Kingdom	14 (9.7)	[110-123]
	China	11 (7.6)	[124-134]
	Canada	11 (7.6)	[135-144]
	Netherlands	7 (4.8)	[145-151]
	New Zealand	6 (4.1)	[152-157]
	Germany	4 (2.8)	[158-161]
	Finland	3 (2.1)	[162-164]
	Sweden	3 (2.1)	[19,165,166]
	Austria	2 (1.4)	[167,168]
	India	2 (1.4)	[169,170]
	Iran	2 (1.4)	[30,171]
	Italy	2 (1.4)	[172,173]
	Singapore	2 (1.4)	[174,175]
	Switzerland	2 (1.4)	[176,177]
	Brazil	1 (0.7)	[178]
	Colombia	1 (0.7)	[179]
	Czech Republic	1 (0.7)	[180]
	Denmark	1 (0.7)	[181]
	France	1 (0.7)	[182]
	Indonesia	1 (0.7)	[183]
	Ireland	1 (0.7)	[184]
	Japan	1 (0.7)	[185]
	Latvia	1 (0.7)	[186]
	Lithuania	1 (0.7)	[187]
	Malaysia	1 (0.7)	[188]
	Poland	1 (0.7)	[189]
	Portugal	1 (0.7)	[190]
	South Korea	1 (0.7)	[191]
	Tunisia	1 (0.7)	[192]
	United States and Canada	1 (0.7)	[193]
**Research method**			
	Randomized controlled trial	81 (55.9)	[19,52,53,55,56,58,62-64,67,68,71,75,77,81-87,89,92-95,101,104-107,109,110,117,118, 121,123-127,129,131-134,136,138-141,144,145,149,153,155,157-159,161,164,166-169, 171,172,174,176-179,181,184,185,187,189,191-194]
	Pilot trial	19 (13.1)	[61,65,69,73,78-80,90,96-98,100,103,108,113,115,148,156,190]
	Feasibility study	16 (11.0)	[57,59,72,91,102,111,114,119,122,130,152,165,175,180,183,186]
	Longitudinal study	5 (3.4)	[76,99,112,150,151]
	Open trial	3 (2.1)	[54,74,154]
	Quasiexperimental study	3 (2.1)	[30,163,188]
	Pre-post study	2 (1.4)	[162,170]
	Other study types^a^	16 (11.0)	[4,60,66,70,88,116,120,128,135,142,143,146,147,160,173,182]
**Year of publication**			
	2019	15 (10.3)	[19,30,71,72,74,106,109,118,119,132,140,155,168,171,193]
	2020	27 (18.6)	[63,67,69,70,76-78,96-100,108,111,117,120-122,139,153,162,164,166,179,180,184,191]
	2021	23 (15.9)	[65,66,73,79-82,107,112,113,123,134,141,142,148,154,156,161,163,169,170,172,173]
	2022	29 (20.0)	[62,68,83-85,87,88,101,114,115,124,126,130,131,133,138,143,144,149,151,157,165,174,175, 182,186,187,192,194]
	2023	33 (22.8)	[52,54,57,58,60,61,75,86,89-91,93-95,102,104,105,110,116,125,129,137,146,147,150,152,158, 160,177,178,181,189,190]
	2024^b^	18 (12.4)	[53,55,56,59,64,92,103,127,128,135,136,159,167,176,183,185,188]

^a^Other study types included: 3-staged participatory, co-design approach; evaluation study; feasibility and acceptability study; implementation-effectiveness study; microrandomized trial; mixed methods study; nonrandomized controlled trial; proof-of-concept study; quantitative study; randomized trial; randomized dismantling trial; randomized factorial trial; repeated measures within-subjects study; secondary data analysis; type 1 effectiveness-implementation randomized controlled trial; and uncontrolled trial.

^b^Until June 7, 2024.

#### Participant Numbers

The number of participants recruited to the experimental groups varied greatly across all studies, ranging from 8 to 2222 (mean 167, SD 303). A total of 88 (60.7%) studies included at least one control group, and the number of participants recruited in these cohorts ranged from 12 to 2355 (mean 148, SD 281).

#### Outcome Measures

The most common primary outcomes assessed across studies were depression (72/145, 49.7%), anxiety (62/145, 42.8%), stress (38/145, 26.2%), well-being (25/145, 17.2%), and mindfulness (11/145, 7.6%). The top secondary outcomes were anxiety (30/145, 20.7%), depression (22/145, 15.2%), well-being (12/145, 8.3%), self-efficacy (10/145, 6.9%), and attributes of mindfulness (11/145, 7.6%). The most frequently used validated mental health scales across studies to assess outcomes are shown in Table 3.

**Table 3:** Most frequently used mental health scales across studies.

Scale	Short name or scale variations used	Studies (N=145), n (%)
Patient Health Questionnaire	PHQ-9, PHQ-8, PHQ-4, and PHQ-2	35 (24.1)
Generalized Anxiety Disorder	GAD-7, GAD-2, and GAD-Q	24 (16.6)
Depression Anxiety Stress Scale	DASS-21	17 (11.7)
Perceived Stress Scale	PSS-10 and PSS-4	16 (11.0)
Warwick Edinburgh Mental Wellbeing Scale	WEMWBS and SWEMWBS	10 (6.9)

#### Incentives

Over half of the studies (80/145, 55.2%) reimbursed participants for taking part in the study or gave some type of incentive for taking part. Reimbursement typically included monetary incentives, such as a voucher or money for completing the baseline, postintervention, and follow-up measures (47/145, 32.4%); some form of course credit for those who were studying (11/145, 7.6%); a voucher, money/gift card, or course credit (11/145, 7.6%); entry into a prize draw (4/145, 2.8%); and a voucher or money and entry into a prize draw (4/145, 2.8%). Other incentives included annual app subscriptions (1/145, 0.7%), a service and data plan and phone for the intervention duration (1/145, 0.7%), and a gift card for each questionnaire completed along with free app membership (1/145, 0.7%).

### Characteristics of Interventions

Ten interventions were used across more than one study, including *Calm* [71,120], *EMIcompass* [148,158], *Grow It!* [150,151], *imi* [75,85], *LifeBuoy* [94,101], *Mindfulness Virtual Community* [139,141], *Spark* [55,93], *Step-by-Step* [128,130], *Tita* [162,164], and *Whitu* [156,157]. Thus, this section of the results reports on all unique interventions (n=135) after excluding duplicate interventions.

#### Target Area

Most interventions were targeted at general mental well-being, depression, anxiety, or stress, or combinations of these 4 areas relating to mental health (Table 4).

**Table 4:** Target area for digital mental health interventions across all studies.

Target area^a^	Value (N=135), n (%)
General mental well-being	52 (38.5)
Depression	19 (14.1)
Anxiety and depression	8 (5.9)
Stress	6 (4.4)
Distress	5 (3.7)
Anxiety	4 (3.0)
LGBTQ+^b^ specific	3 (2.2)
Help-seeking	3 (2.2)
Loneliness	3 (2.2)
Anxiety, depression, and general well-being	2 (1.5)
Anxiety, depression, and stress	2 (1.5)
Anxiety, depression, and suicidal ideation	2 (1.5)
Anxiety and stress	2 (1.5)
Resilience	2 (1.5)

^a^Other target areas not listed in the table: anxiety, depression, and low mood; anxiety, depression, and repeated negative thinking; anxiety, depression, social anxiety, and insomnia; childhood adversity and low self-esteem; distress and adjustment; general well-being; general well-being and substance use; mental health diagnostic and severity spectrum and stages of treatment; mental health literacy; parents have a mental illness or substance use disorder; perfectionism; psychological flexibility; posttraumatic stress disorder and complex posttraumatic stress disorder; reward responsiveness; self-efficacy; self-harm; social anxiety; social connection; stress and general well-being; suicidal ideation; suicidal ideation and insomnia; suicidal ideation, low mood, and self-harm; suicide prevention.

^b^LGBTQ+: lesbian, gay, bisexual, transgender, and queer.

#### Types of Interventions

The most common type of digital tool was an app (51/135, 37.8%), followed by a web-based resource (45/135, 33.3%), a website (19/135, 14.1%), a chatbot (6/135, 4.4%), a virtual reality system (2/135, 1.5%), an app with a game (2/135, 1.5%), and an app with a chatbot (2/135, 1.5%). Other types included an app with a game, an app with text messaging, an app with virtual reality, an app with a wearable, email, a game, metaverse, text messaging, a website with social media, a website with text messaging, and a wearable.

#### Features of Interventions

Table 5 presents the features across all 135 unique digital interventions. In some cases, there were different variations of the same intervention suites, including IntelliCare (Pocket Helper, Purple Chill, and Slumber Time or IntelliCare for College Students) [72,78], MOST+ and MoST-MH [82,97], and Silvercloud (Space for Resilience/Space from COVID-19 or Space from Depression).

**Table 5:** Intervention features across all 135 unique digital interventions.

Feature	Value (N=135), n (%)
Psychoeducation or education	97 (71.9)
Mindfulness, breathing, or relaxation exercises	70 (51.9)
Videos	58 (43.0)
Mood logging or check-in	53 (39.3)
Audio	37 (27.4)
Photos	32 (23.7)
Messaging system or texts	29 (21.5)
Writing exercises, diaries, or journaling	26 (19.3)
Goal setting	26 (19.3)
Interactive tasks	25 (18.5)
Push notifications or reminders	25 (18.5)
Signposting to external resources	24 (17.8)
Gamification or rewards	21 (15.6)
Self-help information or monitoring	19 (14.1)
Homework exercises	18 (13.3)
Personalization or customization	18 (13.3)
Email prompts	16 (11.9)
Coping strategies	14 (10.4)
Personal or peer stories or testimonials	14 (10.4)
Ecological momentary assessment or experience sampling method	12 (8.9)
Gratitude	11 (8.1)
Feedback to users	11 (8.1)
Peer forum or interactions	10 (7.4)
Self-help plan or relapse plan	9 (6.7)
Quiz	8 (5.9)
Video conferencing	6 (4.4)
Virtual reality	4 (3.0)
General health tracking	2 (1.5)
Dot probe	2 (1.5)
Keyword triggers	2 (1.5)

#### Intervention Approach

**Figure 2:**
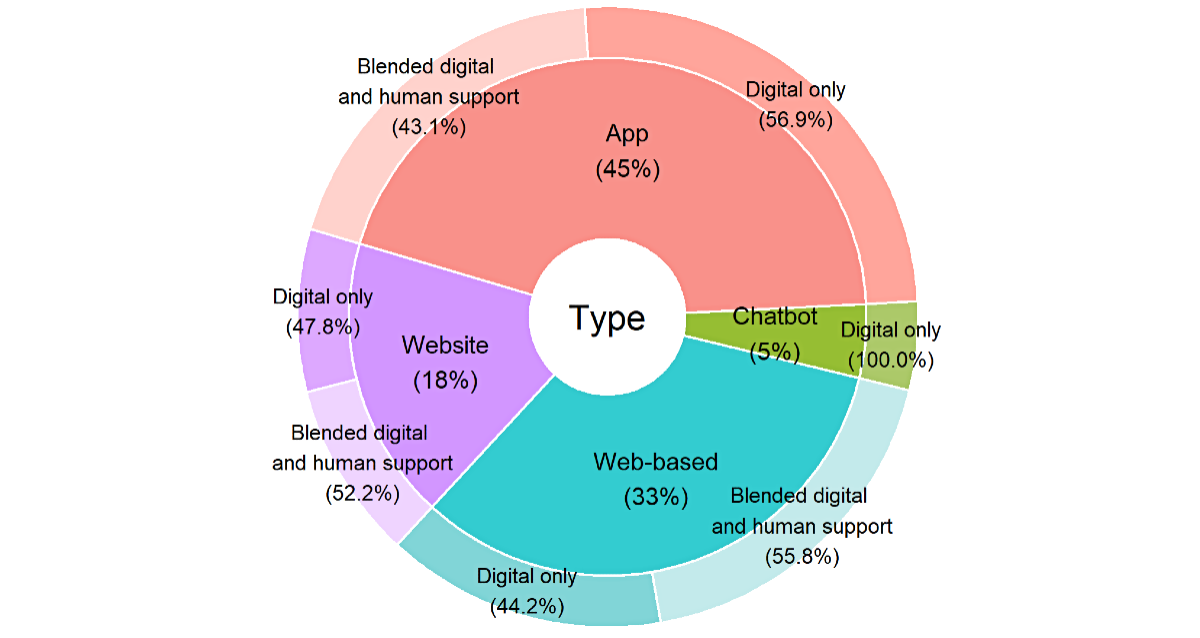
Delivery of interventions across different types of digital tools (N=135).

Just over half of the interventions (70/135, 51.9%) used a digital-only approach, and the rest (65/135, 48.1%) used a blended approach, where participants had some degree of human support (peer, clinical, or research team support) alongside a digital intervention (Figure 2).

**Figure 3:**
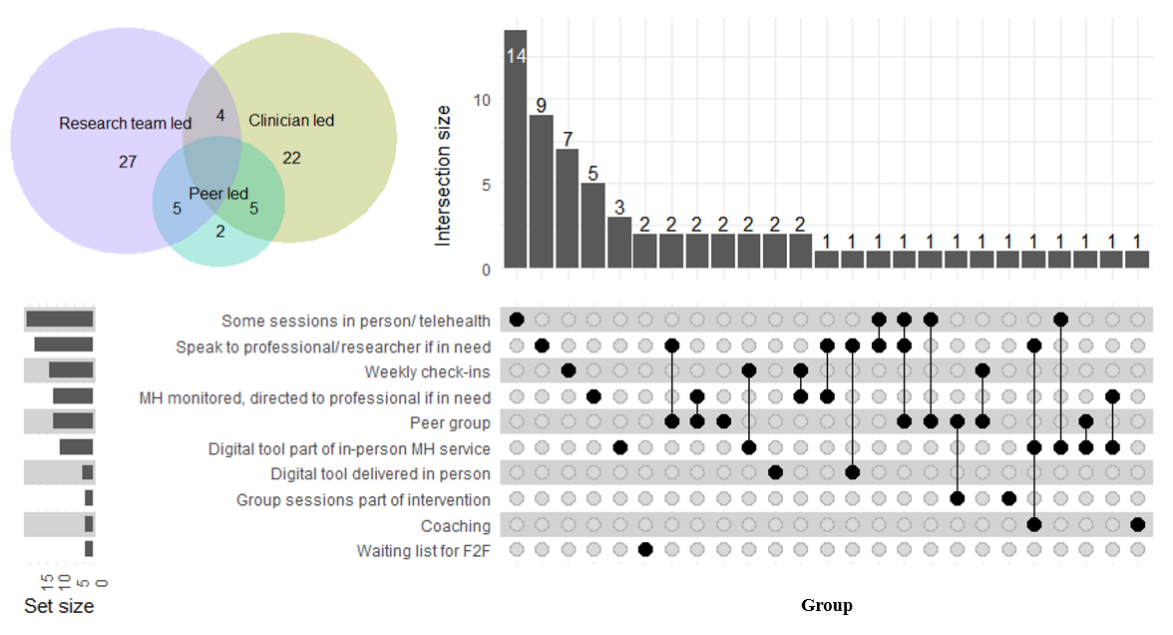
Types of blended interventions used across studies (N=65). Venn diagram showing overlap between peer, clinician, and research team support. Upset plot showing the degree of overlap between specific types of blended support. F2F: face-to-face; MH: mental health.

Of the 65 studies that used some degree of human support, the blended approach between digital and human support varied among studies and overlapped in many cases (Figure 3). The approaches were as follows: some sessions were delivered in person or via telehealth communication (18/65, 27.7%); participants were able to speak to a professional or someone from the research team if needed (16/65, 24.6%); weekly check-ins were held with participants (12/65, 18.5%); the mental health of participants was monitored, and they were directed to professional help if needed (11/65, 16.9%); participants had peer group support (11/65, 16.9%); participants were already attending a mental health service, and the digital tool was a part of the intervention (9/65, 13.8%); the digital tool was delivered in person (3/65, 4.6%); group sessions were delivered as part of the intervention (2/65, 3.1%); coaching from a professional or a member of the research team (2/65, 3.1%); and participants received the digital intervention while on a waiting list for face-to-face services (2/65, 3.1%).

### Target Populations

#### Recruited Populations

Less than half of the studies (63/145, 43.4%) recruited participants from the general population with no specific focus on recruiting young people experiencing mental ill health, 26.2% (38/145) recruited participants experiencing mental ill health symptoms, and 18.6% (27/145) recruited participants who had a mental health diagnosis or who met the clinical or diagnostic criteria for a mental health diagnosis. Moreover, a small number of studies (6/145, 4.1%) included a mix of participants who were not experiencing mental health symptoms and those with a mental health diagnosis, 3.4% (5/145) recruited participants with a specific physical health diagnosis, 2.8% (4/145) recruited only an LGBTQ+ population, and 1.4% (2/145) recruited only a homeless population. Further details on the mental health conditions of those who had a mental health diagnosis or met the diagnostic criteria can be found in Table 6.

**Table 6:** Participant information for studies that recruited individuals who had a mental health diagnosis or met the diagnostic criteria (27 studies).

Mental health condition and details		Sample size (n) of those who started the study, mean (SD)	Percentage (%) of those who completed the study, mean (SD)	References
**Depression (n=7)**		43 (23)	81 (19)	
	Depression (n=3), major depressive disorder (n=3), and young people with or at an elevated risk of depression (n=1)			[57,87,113,122,126,130,135]
**Anxiety (n=5)**		105 (94)	64 (24)	
	Generalized anxiety disorder (n=3), anxiety (n=1), and social anxiety (n=1)			[52,68,132,144,169]
**Depression or anxiety (n=5)**		49 (43)	78 (22)	
	Anxiety or depression (n=4) and clinical levels of depression or anxiety in the Patient Health Questionnaire-8 or Generalized Anxiety Disorder-7 (n=1)			[69,95,119,121,133]
**Depression or anxiety with other co-morbidities (n=5)**		28 (14)	82 (15)	
	Major depressive disorder, reactive attachment disorder, panic disorder, attention-deficit/hyperactivity disorder, intermittent explosive disorder, borderline personality disorder, and parent-child relational problem (n=1)			[148]
	Depression or distress (n=1)			[100]
	Depression/major depressive episode and co-mobilities with generalized anxiety disorder, social anxiety disorder, panic disorder, and agoraphobia (n=1)			[19]
	Anxiety, depression, social anxiety, or insomnia (n=1)			[76]
	Depression, bipolar disorder, anxiety disorder, and psychosis (n=1)			[158]
**Other (n=5)**		42 (26)	75 (19)	
	Psychotic disorder, schizophrenia, schizoaffective disorder, and schizophreniform disorder (n=1)			[108]
	Posttraumatic stress disorder and complex posttraumatic stress disorder (n=1)			[187]
	Social phobia (n=1)			[96]
	Psychosis and borderline personality disorder (n=1)			[103]
	Specific details not reported; inclusion criteria stated that only participants with a current mental health diagnosis documented in their electronic medical record or those who received mental health services within 3 months according to a self-report or a report by a parent or clinician could participate (n=1)			[82]

#### Sampling and Recruitment Strategies

**Figure 4:**
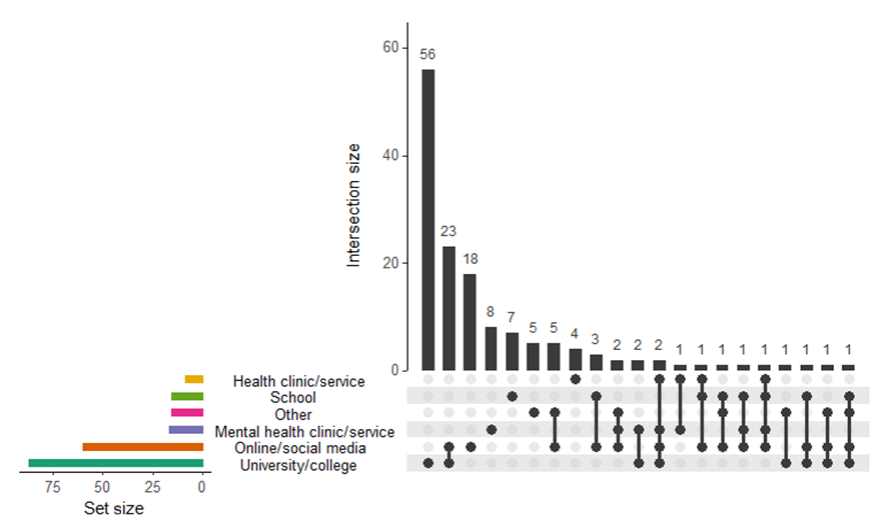
Participant recruitment sources across all studies (N=145).

Almost all of the studies (140/145, 96.6%) used a convenience sampling approach, while the remaining studies used a convenience sampling approach with snowballing (1/145, 0.7%), cluster randomization of schools (1/145, 0.7%), geographically representative sampling (1/145, 0.7%), sampling representative of the depressive population (1/145, 0.7%), and random sampling (1/145, 0.7%). Participants were predominately from educational institutions, as universities were the most popular source for recruitment (87/145, 60.0%), followed by online or social media (60/145, 41.4%) (Figure 4).

#### Age and Gender Identity

The overall mean age of participants in the experimental groups from all studies that reported this variable (n=133) was 20.8 years. A total of 12 studies did not mention the specific ages of their populations, however the age range of the experimental groups was 16-25 years. The overall mean age of participants in the control groups from all studies that reported this variable (n=82) was 20.8 years. A total of 6 studies only reported the age range.

**Figure 5:**
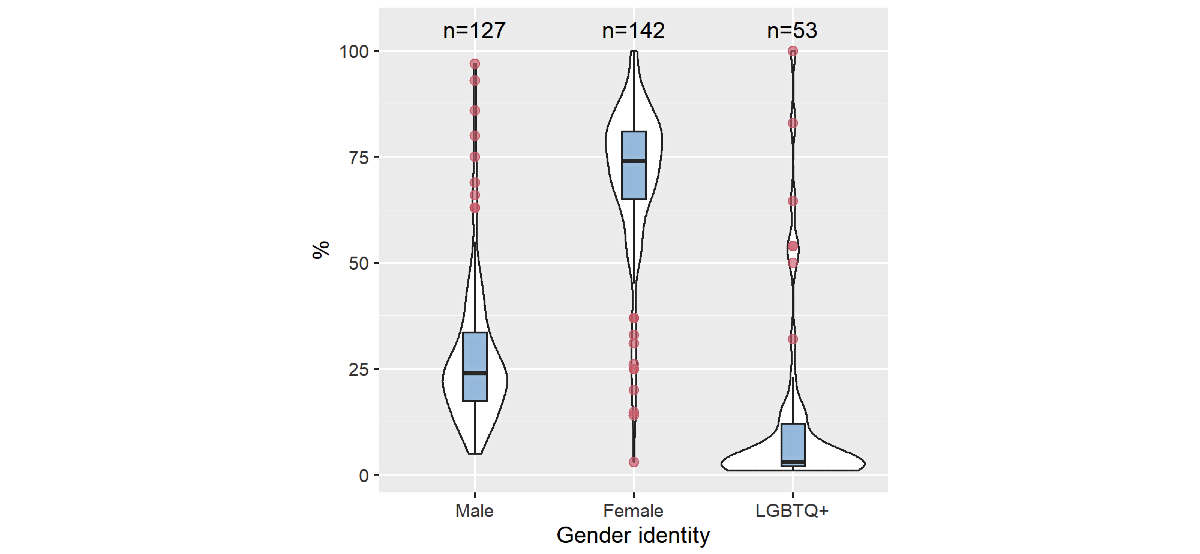
Gender split in experimental groups across all studies (N=145). LGBTQ+: lesbian, gay, bisexual, transgender, and queer.

Females were largely overrepresented within the study groups, making up 71% of study populations on average across 142 studies, while males on average made up 28% of study populations across 127 studies (Figure 5). Three studies included an entirely female population [70,166,171]. Gender was not reported equally across studies, as 15 studies did not include or report the gender breakdown of male participants and 3 did not include or report the gender breakdown of female participants. Only 53 (36.6%) studies included individuals identified as LGBTQ+ (Figure 5), with the highest representation from transgender and nonbinary individuals. In these studies, LGBTQ+ participants represented 1% to 100% of the study population (mean 13%, SD 22%). As mentioned in Table 4, 3 interventions were specifically targeted at improving the mental health of those who identified as LGBTQ+.

#### Marginalized Groups

**Figure 6:**
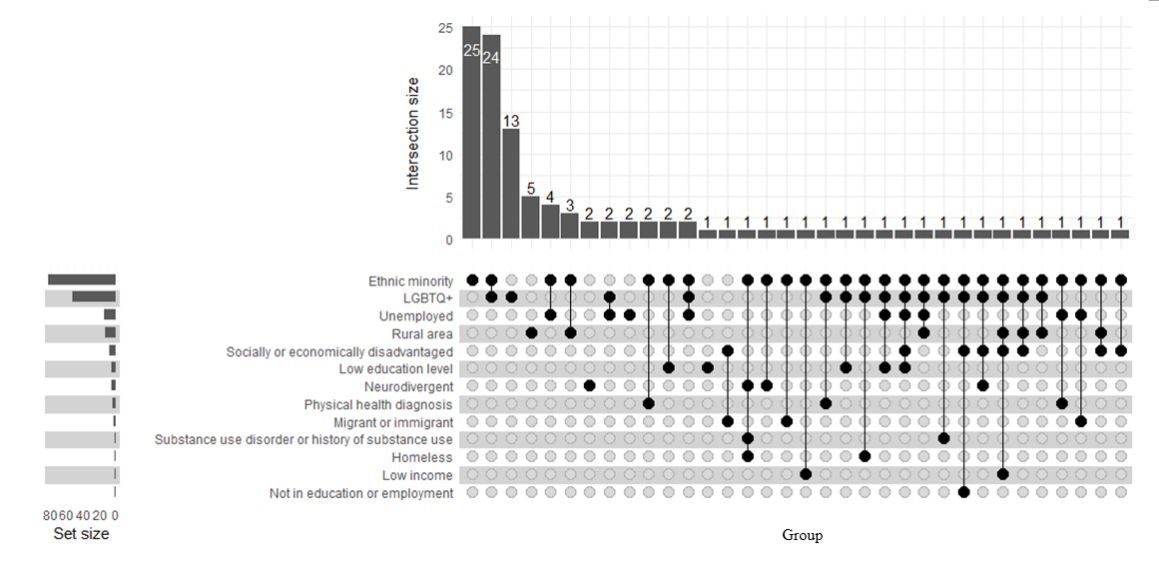
Marginalized groups represented across all studies (N=145). LGBTQ+: lesbian, gay, bisexual, transgender, and queer.

Three-quarters (108/145, 74.5%) of all studies included marginalized groups in their study populations (Figure 6). Minority ethnic groups were most frequently represented, with 82 (56.6%) studies including participants from a variety of ethnic groups (Figure 6). LGBTQ+ young people were the second most represented group (53/145, 36.6%) (Figure 6). Only a very small proportion of studies had other groups, including those who were unemployed (15/145, 10.3%), were living in a rural area (13/145, 9.0%), were socially or economically disadvantaged (8/145, 5.5%), had a low education level (6/145, 4.1%), were neurodivergent (5/145, 3.4%), were living with a physical health condition (4/145, 2.8%), were migrants or immigrants (3/145, 2.1%), had a low income (2/145, 1.4%), were homeless (2/145, 1.4%), had current or previous substance use issues (2/145, 1.4%), and were not educated or employed (1/145, 0.7%).

#### Intervention Approach Across Main Populations

**Figure 7:**
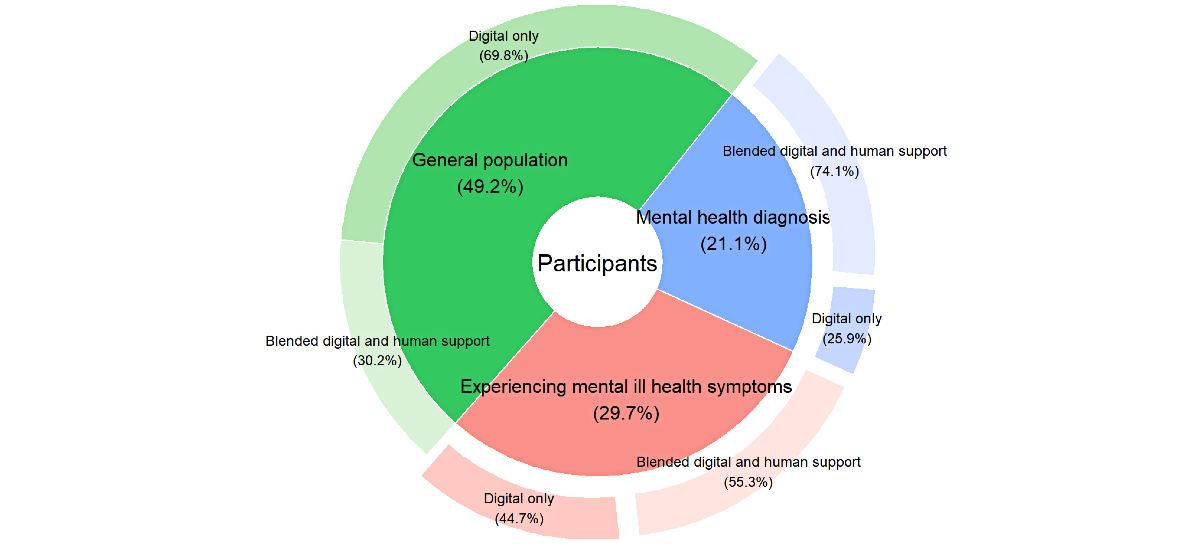
Breakdown of digital-only support versus blended human and digital support.

Most studies using interventions targeted at the general population (n=63) adopted a digital-only approach (70%), whereas studies that recruited people experiencing mental ill health symptoms (n=38) had a higher percentage of blended support (54%) and those that recruited people with a mental health diagnosis (n=27) had the highest proportion of blended human and digital support (74%) (Figure 7). The type of blended approach varied across study populations, with researcher team support being the most common approach in the general population (12/19, 63.2%). In study populations of those with mental ill health symptoms, research team (9/21, 42.9%) and clinician support (9/21, 42.9%) were equally used. For those with a mental health diagnosis, clinician support was the most common (14/20, 70.0%).

#### Modalities

**Figure 8:**
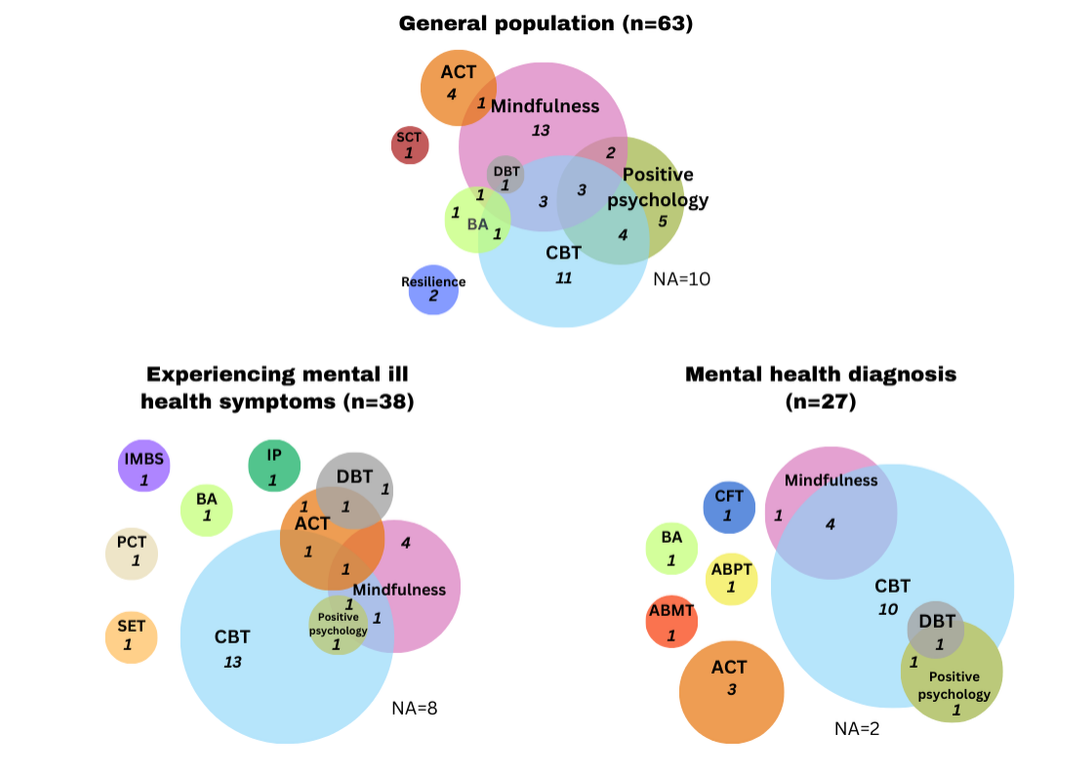
Treatment modalities used in digital mental health interventions across main participant groups. ABMT: attention bias modification training; ABPT: affect-based psychodynamic therapy; ACT: acceptance and commitment therapy; BA: behavioral activation; CBT: cognitive behavioral therapy; CFT: compassion-focused therapy; DBT: dialectical behavior therapy; IMBS: information-motivation-behavioral skills; IP: interpersonal psychotherapy; NA: not applicable; PCT: perceptual control theory; SCT: social cognitive theory; SET: self-efficacy training.

Many of the interventions reported in the studies used multiple treatment modalities, and these differed depending on the population group (Figure 8). The most common modality was CBT, which was used in interventions across 63 (43.4%) studies, with 39 (26.9%) using CBT alone and 24 (16.6%) using CBT in combination with another modality. The second most common treatment modality was mindfulness, which was used in interventions across 40 (27.6%) studies, with 21 (14.5%) using mindfulness alone and 19 (13.1%) using mindfulness in combination with another treatment modality. The third most popular type was positive psychology, which was used in 21 (14.5%) studies, with 7 (4.8%) using positive psychology as the sole modality and 14 (9.7%) using it in combination with another treatment modality.

### Intervention Features

**Figure 9:**
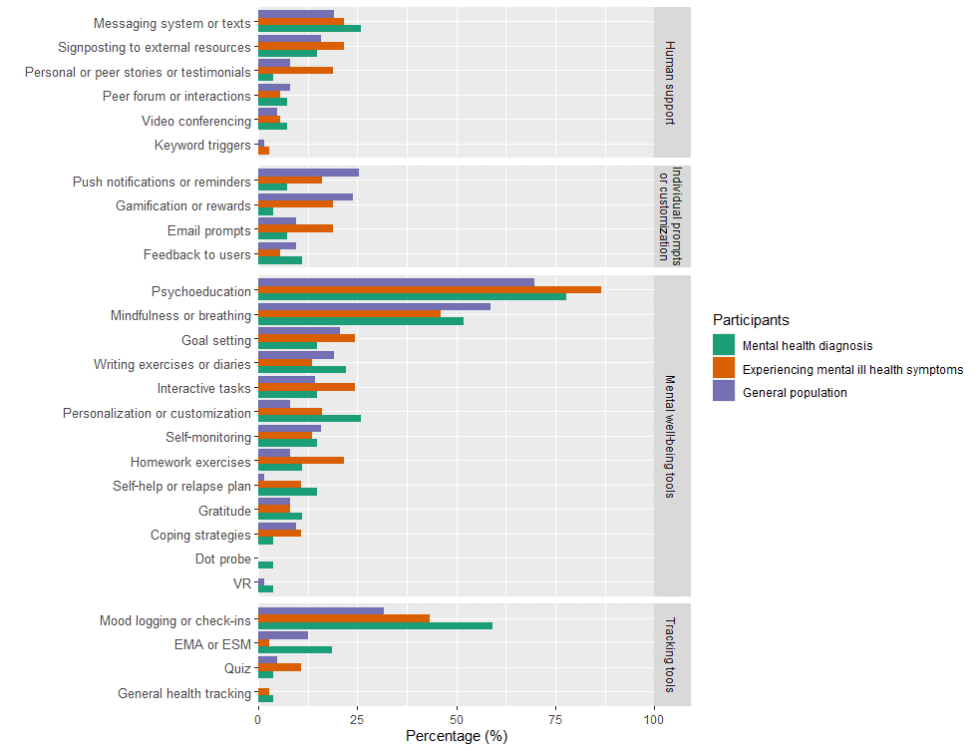
Intervention features across most common populations. EMA: ecological momentary assessment; ESM: experience sampling method; VR: virtual reality.

In studies that recruited participants from the general population (n=63), the most common features used in digital interventions were psychoeducation or education (44/63, 70%), mindfulness or breathing exercises (37/63, 59%), and mood logging (20/63, 32%) (Figure 9).

In studies that recruited people experiencing mental ill health symptoms (n=38), the top features in digital interventions were the same, including psychoeducation or education (32/38, 86%), mindfulness or breathing exercises (17/38, 45%), and mood logging (16/38, 43%) (Figure 9).

In studies that recruited people with a mental health diagnosis or those who met diagnostic criteria (n=27), the most common features in digital interventions were psychoeducation or education (21/27, 78%); mood logging, check-in, or self-monitoring of symptoms (16/27, 59%); and mindfulness or breathing exercises (5/27, 25%) (Figure 9).

Personalization or customization was a more common feature in interventions for people experiencing mental ill health symptoms (6/38, 16%) or those with a mental health diagnosis (7/27, 26%) compared to the general population (5/63, 8%) (Figure 9). Self-help plans or relapse plans were used more commonly in people experiencing mental ill health symptoms (4/38, 10%) or those with a mental health diagnosis (4/27, 15%) compared to the general population (1/63, 1%) (Figure 9). Gamification or reward incentives were used more often in interventions for the general population (15/63, 24%) and for people experiencing mental ill health symptoms (7/37, 19%) compared to interventions for those with a mental health diagnosis (1/27, 4%) (Figure 9).

### Retention

Retention rates ranged from 16% to 100%, with a mean retention rate of 66% (SD 23%) across all studies. Table 7 highlights the retention rate across study type, delivery, type of digital tool, study population, and incentive versus no incentive.

**Table 7:** Retention rates across study characteristics.

Characteristic		Participants (n) who started the study		Participants (%) who completed the study	
		Range	Mean (SD)	Range	Mean (SD)
**Study type**					
	Randomized controlled trial (n=81)	8-2222	177 (305)	16-100	70 (22)
	Pilot study (n=19)	8-630	69 (140)	24-100	79 (22)
	Feasibility study (n=16)	10-692	77 (167)	19-100	66 (24)
**Delivery**					
	Digital only (n=77)	8-2327	189 (370)	19-100	69 (23)
	Digital intervention with human support (n=68)	8-2222	165 (319)	16-100	71 (23)
**Type of digital tool**					
	App (n=58)	10-2222	121 (294)	33-100	76 (18)
	Web-based (n=43)	10-810	176 (207)	19-100	64 (24)
	Website (n=23)	12-1982	258 (436)	16-100	57 (25)
**Study population**					
	General population (n=63)	10-2327	263 (444)	19-100	68 (23)
	Experiencing mental ill health symptoms (n=38)	8-1982	166 (328)	16-100	64 (22)
	Mental health diagnosis (n=27)	11-280	53 (56)	32-100	76 (22)
**Incentive**					
	No incentive given (n=65)	8-2222	193 (382)	16-100	65 (24)
	Any incentive given (n=80)	8-2327	166 (318)	24-100	73 (21)

#### Studies With High Retention Rates

A total of 36 studies had very high retention rates of 90%-100% [57,61,66,67,69,73,75,77-79,82,84,85,88,95,100,103,107, 114,124-126,131-133,135,141,155,160,163,167,172,175,176,185,190]. These studies recruited between 8 and 222 participants (mean 60, SD 52). The approach ranged from a single, one-off session to a 24-week trial period, with an average length of 6 weeks. Only 10 of these 36 studies recorded any follow-up measures, anywhere from 2 weeks after the study up to 12 weeks, with an average follow-up duration of 8.5 weeks. Approximately two-thirds (22/36, 61%) of these studies gave participants some form of reimbursement, such as financial incentives or course credits for students, for completing the study.

## Discussion

### Principal Findings

This study sought to investigate the literature on DMHIs for young people aged 16-25 years in order to assess (1) the characteristics of studies, (2) the characteristics of DMHIs offered to young people and the level of human support in combination with digital mental health, (3) the target populations of DMHIs for young people, and (4) the retention rates across studies involving DMHIs for young people.

Studies were spread out globally, mostly originating from the United States of America (43/145, 29.7%), Australia (16/145, 11.0%), and the United Kingdom (14/145, 9.7%). There was a range of study types (mostly RCTs: 81/145, 55.9%), and the majority of studies had a control group (88/145, 60.7%). Just under half of the studies (65/145, 44.8%) focused on treatment, and the rest (80/145, 55.2%) focused on mental health promotion or prevention of mental health problems. The mean prescribed length of using an intervention was 6 weeks, and 43.4% (63/145) of studies recorded follow-up data after the intervention period, most commonly at 12 weeks. Participant numbers varied greatly across studies from 8 to 2222, with a mean of 167 but with a large SD of 303. Over half of the studies (80/145, 55.2%) provided reimbursements for study participation, and these were mostly in the form of financial incentives.

Interventions mostly targeted general mental well-being, depression, anxiety, or stress. Apps (51/135, 37.8%), web-based resources (45/135, 33.3%), and websites (19/135, 14.1%) were the most popular digital tools. Psychoeducational content (97/135, 71.9%) and mindfulness or breathing exercises (70/135, 51.9%) were most common across digital interventions. Moreover, 48.1% (65/135) of interventions involved a blended approach combining human support with a digital intervention, while 51.9% (70/135) involved a digital-only approach.

The largest group of participants across studies included young people from the general population without reported mental health issues (63/145, 43.4%). The next most common group included those experiencing mental ill health symptoms (38/145, 26.2%), followed by those with a mental health diagnosis or meeting diagnostic criteria (27/145, 18.6%). Among those diagnosed, most had anxiety or depression; 5 studies included participants with depression or anxiety along with other co-morbid mental health conditions and 5 studies included participants with other mental health conditions. Convenience sampling was prevalent (140/145, 96.6%), with universities and colleges being the primary recruitment location. Three-quarters of the studies (108/145, 74.5%) included young people from marginalized groups. Digital-only approaches were common for the general population, while blended human-digital support was more often used for those with mental ill health symptoms or a mental health diagnosis. CBT, positive psychology, and mindfulness were the most popular approaches in interventions for the general population, whereas digital interventions for mental health symptoms or diagnosis often used CBT or other specific therapeutic modalities. Gamification or reward incentives were more prevalent in interventions for the general population (15/63, 24%) and populations experiencing mental health symptoms (7/37, 19%) and less prevalent in interventions for individuals with mental health diagnoses (1/27, 4%). The mean age of participants was 20.8 years across all studies. Females were largely overrepresented (71% of study populations), while males typically constituted 30% of study populations. Only 36.6% (53/145) of studies included LGBTQ+ participants, including transgender and nonbinary individuals. In these studies, LGBTQ+ representation averaged 13%.

The average retention rate across all studies was 66% (SD 23%). Retention rates were higher than the mean for pilot studies (mean 79%), apps (mean 76%), studies that recruited participants with a mental health diagnosis (mean 76%), and studies that offered any type of incentive (mean 73%).

### Limitations

In this review, some information was missing during data extraction as indicated in the results. For example, 12 studies reported age ranges instead of average age, but most data were largely reported. As a scoping review methodology was employed, a risk of bias assessment or an assessment of the efficacy or effectiveness of DMHIs was not conducted. As a quality assessment was not conducted, the interpretation of findings regarding retention rates might be affected. In particular, 44% of studies were not RCTs, which are considered the gold standard in research for evaluating effectiveness. Therefore, the finding that retention rates for pilot studies were higher (79% on average) than those for RCTs (70% on average) should be interpreted with caution. Given the heterogeneity of DMHIs included in this study, a systematic review and meta-analysis was not feasible, and thus, it was not possible to assess effectiveness. However, future work could focus on specific areas, for example, apps for specific cohorts of young people, to assess effectiveness. Despite these limitations, this scoping review provides extensive insights into the current landscape of DMHIs for young people, ranging from mental health promotion and primary prevention to more targeted treatment interventions, and indicates useful areas for future research.

### Comparison With Prior Work

While roughly half of the studies included in this review focused on the treatment of common mental health problems, future work could direct more resources toward promotion and prevention measures to halt the escalation of mental ill health. The period between 18 and 25 years of age represents a transitional phase marked by increased exposure to risk factors like instability, employment search, identity exploration, and heightened self-focus, elevating the risk of mental health conditions [195]. Thus, for young people, it is important to focus on mental health promotion and prevention strategies, which include universal, whole population approaches irrespective of current mental health status; selective approaches for specific risk factors; indicated approaches that focus on early subclinical symptoms; and tertiary approaches that target specific mental health conditions [196]. A recent meta-analysis found that the use of digital mental health promotion tools among youth aged between 11 and 18 years resulted in small significant improvements in general well-being and small-to-medium significant improvements in anxiety [8]. The current literature indicates that universal approaches used within psychological or psychoeducational interventions may improve symptoms of anxiety but may not prevent depressive or anxiety disorders [197]. Further research into digital mental health promotion for young people would be beneficial to strengthen these findings, given the heterogeneity of studies in this area.

Psychoeducational content and mindfulness or breathing exercises were most commonly used across interventions for all populations, and throughout the studies, there was a major focus on CBT and mindfulness-based approaches. Although CBT is considered the gold standard for treatment, as a previous systematic review and meta-analysis found that digitally delivered CBT interventions had greater effect sizes compared to other modalities [198], future interventions could use a broader spectrum of therapeutic models. Acceptance and commitment therapy, which is another type of third-wave cognitive treatment, was used in a small proportion of studies, and it has been previously demonstrated to be effective in enhancing and sustaining mental health outcomes across various demographics; however, evidence of the clinical significance of these effects is lacking [199]. Given the scarcity of research, a future direction could be to run large-scale trials to determine the effectiveness of other therapeutic modalities outside of CBT or combined approaches, particularly in the digital mental health context. In addition to these therapeutic modalities, there is limited research on theoretical models informing DMHIs, particularly for young people. A commentary paper provided a conceptual overview of how established behavior theories and models, such as Health Belief Model, Theory of Planned Behavior, Transtheoretical Model, and Social Cognitive Theory, can inform the development of a digital intervention for individuals with mental ill health [200]. Naslund et al [200] suggested that individual characteristics should inform intervention design and shape the content, while theory should inform strategies to support behavior change, which can be modified in real-time based on user feedback, and that theory can also guide outcomes to inform behavior mechanisms and intervention modifications. In 2020, the World Health Organization published a framework for planning, developing, and implementing youth-centered DMHIs, which mentions developing a theory-driven approach but does not provide explicit details on how to approach this using psychological theory [201]. Further research is needed on how these theoretical approaches can be adapted for digital delivery and their appropriateness for young people.

One of the most prominent results was the exclusion of more marginalized groups outside of ethnic minorities. However, there may be underreporting of the inclusion of marginalized groups as studies may not always capture this information when collecting participant demographic information. Based on the data used in this review, there was a lack of gender diversity and lack of representation from certain populations, including young people who were unemployed, were living in a rural area, were socially or economically disadvantaged, had a low education level, were neurodivergent, were living with a physical health condition, were migrants or immigrants, had a low income, were homeless, were experiencing current or previous substance use issues, and were not educated or employed. These groups are often excluded from digital mental health studies, as convenience sampling is most often used, with participants most commonly being recruited through universities or colleges. In particular, LGBTQ+ young people often experience victimization and have poorer mental health and higher rates of self-harm and suicidal ideation compared to their cisgender and heterosexual peers [202,203]. Looking more broadly across the whole population in a UK mental health service, a recent study highlighted that certain vulnerable groups, including ethnic minorities, individuals with disabilities, those born outside of the UK, and those with lower academic attainment, were underrepresented in psychological therapies at the national level [204]. Other previous work also identified that more research is needed on preventative DMHIs in young people having poor or underserved backgrounds [205]. It is clear that future work in this area should aim to be more inclusive, with representation from marginalized and vulnerable young people.

In addition, there was a lack of inclusion of a range of mental health conditions outside of anxiety and depression, given that only 5 studies focused solely on study populations of people with diagnoses that did not include anxiety or depression. Previous systematic reviews on digital interventions for young people with anxiety and depression have found small to medium effect sizes when comparing digital interventions, most commonly using CBT modalities, with interventions used in a control group [32,33]. In addition, studies targeting those with mental health diagnoses were relatively underpowered, with sample sizes falling short of the previously reported median of 106 participants in efficacy and effectiveness trials [206].

The average prescribed length of using an intervention was 6 weeks, but there were large variations from a single one-off session to a use period of 6 months, and only 43.4% (63/145) of studies recorded follow-up data after the intervention period at different time points. Compared to traditional therapy, there are no consistent prescribed lengths for DMHIs. Depending on the type of DMHI being delivered, future work could explore intervention lengths akin to that prescribed by psychological therapy services. For example, in the UK, the National Health Service provides “NHS Talking Therapies,” formerly known as Improving Access to Psychological Therapies, which follows a stepped care model. Within this model, individuals experiencing mild-to-moderate mental ill health symptoms (step 2) would receive low-intensity treatment, which could be up to 6 sessions with a mental health professional. There was also a lack of long-term follow-up data in over half of the studies, which would make it difficult to track sustained changes in mental health over time. Future work should look at standardizing the length of treatment within DMHIs and including long-term follow-up data to help determine the efficacy or effectiveness of digital tools compared to traditional face-to-face mental health support.

The average retention rate across all studies was 66%, which falls slightly short of the weighted average of 69% reported for the retention of internet-based mental health interventions [207]. In comparison to other studies in this field, 74.8% of young people aged 11-18 years completed mental health promotion interventions with a digital component [8] and 80% of children and young people used digital health interventions or completed follow-up measures [21]. The average rate identified in this study is also comparable with that in other studies on youth mental health interventions generally that did not involve a digital component. For example, a study that examined interventions for young people at risk of psychosis reported a pooled retention rate of 66% [208]. For long-term clinical studies, a rate of 80% or higher is considered acceptable or high completion [209], so there is still room for improvement. Sustaining engagement with DMHIs in real-world settings outside of RCTs is even more challenging, as highlighted by Fleming et al [210], and only 0.5% to 28.7% of users from the general population completed or continually used self-help interventions for anxiety and depression. Some previous strategies have been identified to recruit young people and keep them engaged in longitudinal health research, such as recruitment through social media, including financial incentives for taking part, joining with peer groups, and incorporating data collection that is user friendly and flexible, and young people also emphasized the importance of social connection with both peers and the research team [211]. Another study that examined barriers and facilitators to engagement with digital health interventions among those aged 2-25 years found that children and young people preferred digital health interventions with features such as videos, limited text, personalization, ability to connect with others, and options to receive text message reminders [21]. An important area for future work will be to identify or incorporate better strategies to improve engagement and completion, particularly for marginalized populations. The main recommendations for the field based on key findings in this review can be found in Table 8, which include general recommendations for future research, as well as relevant findings for clinicians, technology developers, and policy makers.

**Table 8:** Recommendations for future work based on key findings in this review.

Recommendation		Key findings
**General recommendations for future research**		
	Expand research in underrepresented regions: More research is needed in low- and middle-income countries to ensure digital mental health interventions are accessible and culturally relevant across diverse global populations.	The majority of studies were conducted in high-income countries, particularly the United States, Australia, and the United Kingdom.
	Enhance study design and follow-up assessments: Future research should incorporate multiple follow-up assessments to evaluate the sustained impact of interventions beyond the intervention period.	Over half of the studies were randomized controlled trials, but few studies included long-term follow-ups.
	Improve diversity and inclusion in participant recruitment: Efforts should be made to recruit participants from more diverse backgrounds, including ethnic minorities, low-income populations, neurodivergent individuals, and those living in rural areas. Additional demographic information should be recorded to confirm inclusion.	Most studies relied on convenience sampling from universities and online platforms, limiting representation.
	Address gender imbalance in study samples: Aim for a more balanced gender distribution to understand the effectiveness of digital interventions across all genders.	Females were largely overrepresented in study populations (71% on average), and LGBTQ+^a^ young people were only included in 37% of studies overall.
**Recommendations for clinicians**		
	Increase focus on blended human-digital approaches: Blended approaches, particularly for individuals with mental health diagnoses, could be prioritized to improve engagement and outcomes.	While 52% of interventions were fully digital, nearly half (48%) included some form of human support.
	Standardize and validate outcome measures: Greater consistency in validated measures is needed to assess intervention effectiveness reliably.	Studies used a variety of mental health scales, making it difficult to compare results.
	Tailor digital interventions to specific mental health needs: More targeted interventions should be developed to support individuals with severe or complex mental health conditions outside of anxiety and depression.	While general mental well-being was the most common target along with anxiety, depression, and stress, specific conditions, such as posttraumatic stress disorder and psychosis, were underrepresented.
**Recommendations for technology developers**		
	Encourage multicomponent interventions: Combining multiple elements, such as interactive tasks, gamification, and peer support, may enhance engagement.	The most common intervention features were psychoeducation, mindfulness, and mood tracking.
	Leverage emerging technologies for mental health: Future research could explore the potential of advanced technologies to enhance engagement and personalization.	Apps and web-based resources were the dominant modalities, with minimal use of virtual reality, chatbots, or artificial intelligence–driven interventions.
**Recommendations for policy makers**		
	Increase accessibility and affordability: Future interventions could focus on cost-effective solutions that remain accessible without financial incentives.	Over half of the studies provided financial incentives, which may not be sustainable in real-world implementation.
	Promote implementation in real-world settings: Future research should explore how digital tools can be integrated into health care systems, schools, and workplaces for broader reach and long-term sustainability.	Many interventions were tested in research environments but lacked real-world application.

^a^LGBTQ+: lesbian, gay, bisexual, transgender, and queer.

### Conclusions

This scoping review has provided a comprehensive overview of DMHIs for young people aged 16-25 years. It has highlighted the global spread of studies, focusing on the treatment, prevention, and promotion of mental health. The scoping review sheds light on the need for greater inclusivity in research, given the high proportion of females who are often recruited from universities through convenience sampling. The review also highlights a lack of representation from marginalized groups and emphasizes the importance of long-term follow-up data to assess the efficacy and effectiveness of these interventions. Additionally, the review highlights challenges in sustaining engagement and completion, particularly outside of controlled trials, and suggests the importance of incorporating better engagement strategies, especially for marginalized populations. Overall, this review calls for a more inclusive and comprehensive approach to DMHIs for young people.

## Appendix

Multimedia Appendix 1 Total number of results returned after searching 6 databases.

Multimedia Appendix 2 PRISMA-ScR (Preferred Reporting Items for Systematic Reviews and Meta-Analyses extension for Scoping Reviews) checklist.

Multimedia Appendix 3 Intervention duration across studies. The red line indicates the mean study duration.

Multimedia Appendix 4 Data extracted from all 145 studies.

## Abbreviations

CBT: cognitive behavioral therapy
DMHI: digital mental health intervention
LGBTQ+: lesbian, gay, bisexual, transgender, and queer
OSF: Open Science Framework
RCT: randomized controlled trial

## Data Availability

The dataset containing data extracted from the studies included in this review can be found in Multimedia Appendix 4.
