# Supplementary material for: Digital Mental Health Interventions for Young People Aged 16-25 Years: Scoping Review
Source: J Med Internet Res. 2025 May 9;27:e72892. doi: 10.2196/72892 (PMC12102633; doi:10.2196/72892)

**Multimedia Appendix 1.** Total number of results returned after searching 6 databases.

Scopus 🡺


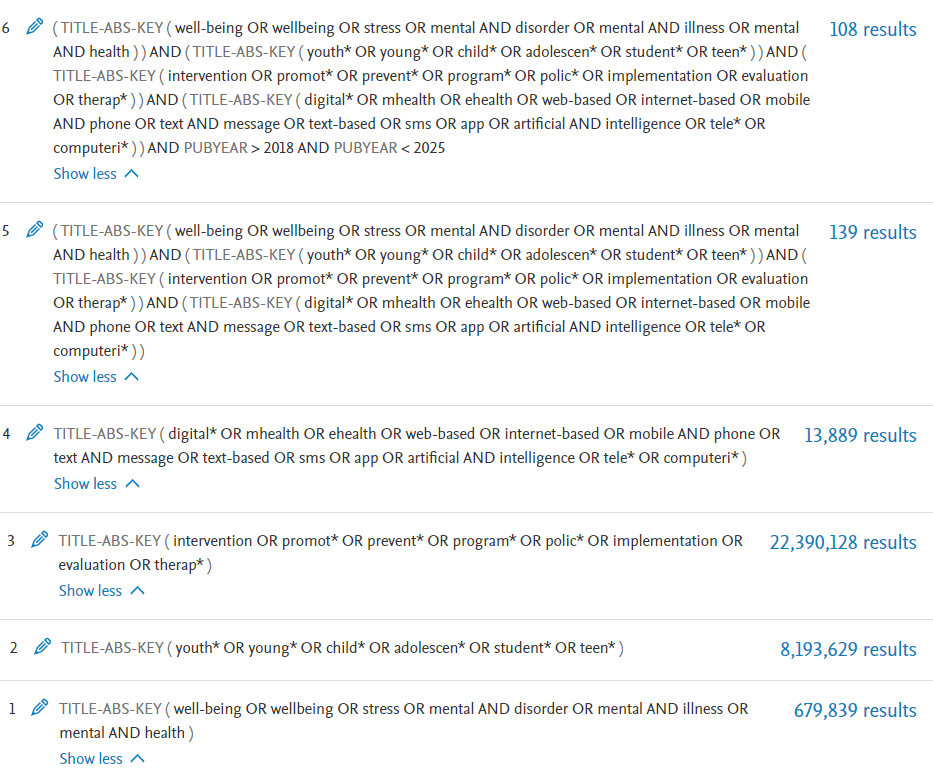


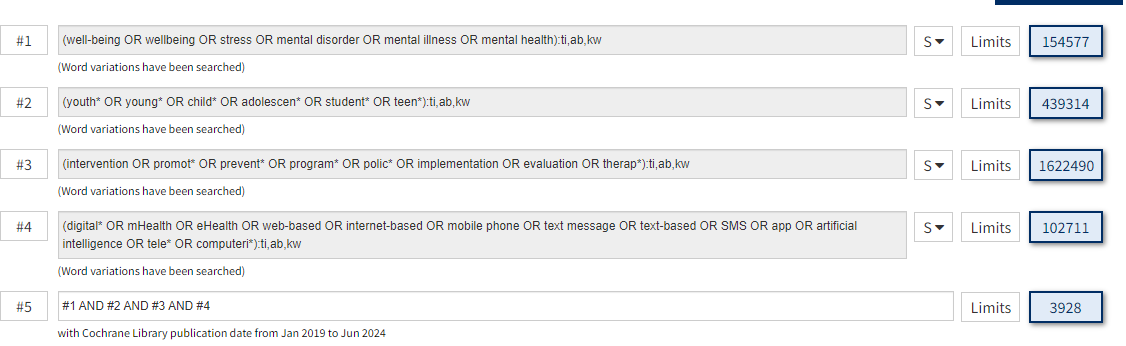
Cochrane 🡺


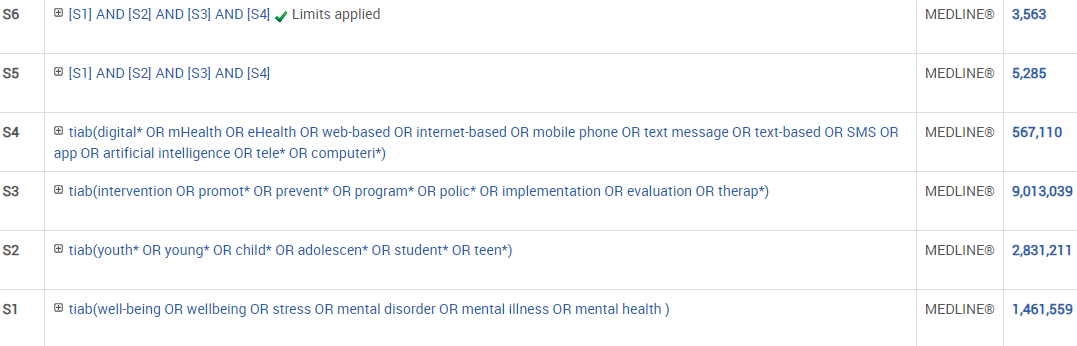
Medline 🡺


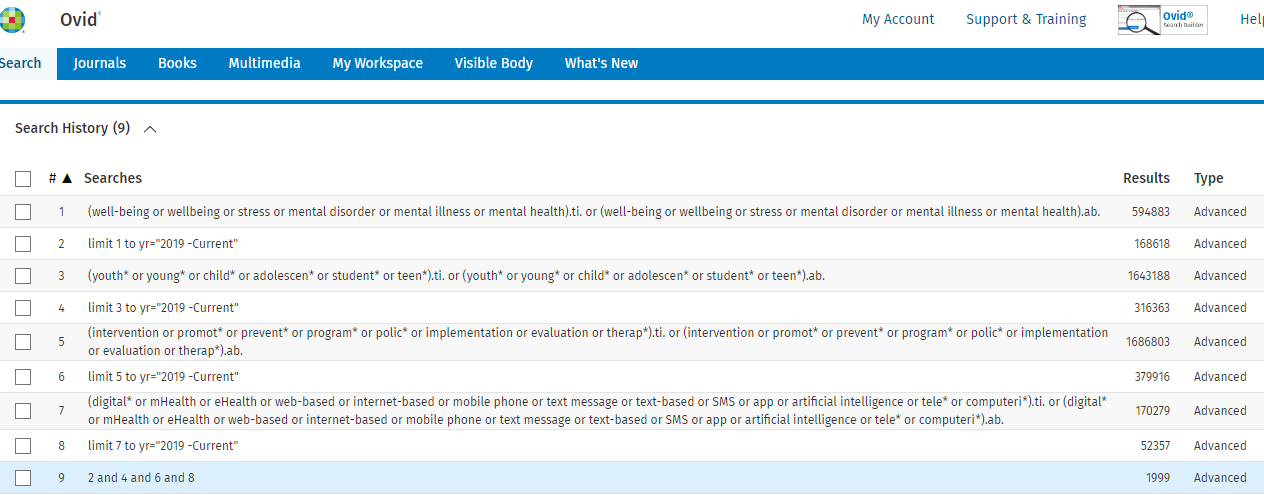
PsychInfo 🡺

Web of Science 🡺


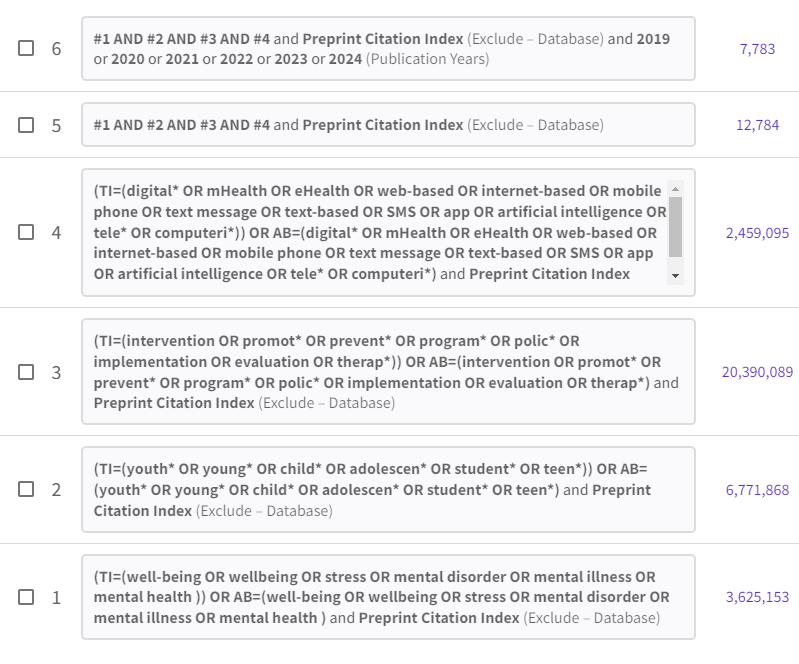

Supplement: Multimedia Appendix 1 [file jmir_v27i1e72892_app1.docx]
