# Supplementary figures and images for: Digital Mental Health Interventions for Young People Aged 16-25 Years: Scoping Review
Source: J Med Internet Res. 2025 May 9;27:e72892. doi: 10.2196/72892 (PMC12102633; doi:10.2196/72892)

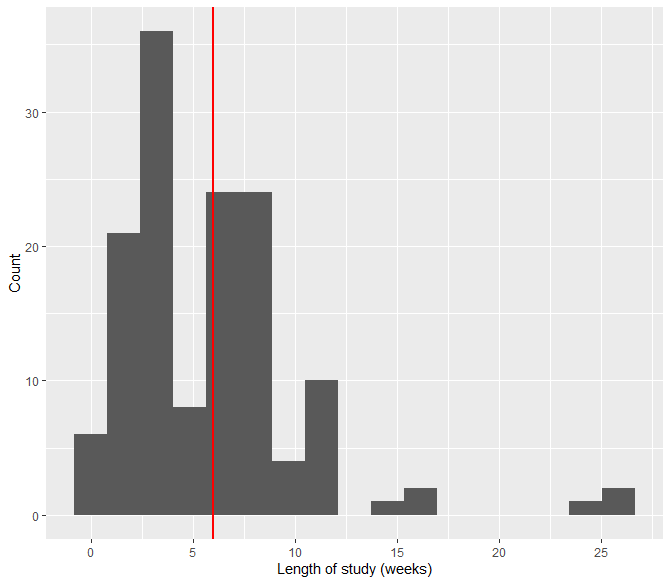

Supplement: Multimedia Appendix 3 [file jmir_v27i1e72892_app3.png]
